# Drug Utilization Evaluation and Impact of Pharmacist Interventions on Optimization of Piperacillin/Tazobactam Use: A Retrospective Analysis and Prospective Audit

**DOI:** 10.3390/antibiotics12071192

**Published:** 2023-07-15

**Authors:** Savera Arain, Fahad Khalawi, Sainul Abideen Parakkal, Hassan S. AlHamad, Shabeer Ali Thorakkattil, Faisal Fahad J. Alghashmari, Bader AlHarbi, Nujud Bakhashwain, Weaam Mustafa Alzawad, Ali AlHomoud

**Affiliations:** Pharmacy Services Department, Johns Hopkins Aramco Healthcare (JHAH), Dhahran 34465, Saudi Arabia; fahad.khalawi@jhah.com (F.K.); sainulabideen.parakkal@jhah.com (S.A.P.); hassan.alhamad@jhah.com (H.S.A.); faisal.alghashmari@jhah.com (F.F.J.A.); bader.alharbi.7@jhah.com (B.A.); 2180006372@iau.edu.sa (N.B.); 2180004799@iau.edu.sa (W.M.A.); ali.homoud.1@jhah.com (A.A.)

**Keywords:** piperacillin/tazobactam, drug utilization evaluation, antibiotic resistance, infection control, antimicrobial stewardship, de-escalation of antibiotics, pharmacist interventions

## Abstract

(1) Background: Piperacillin/tazobactam is a broad-spectrum antimicrobial encompassing most Gram-positive and Gram-negative aerobic and anaerobic bacteria. The inappropriate use of such broad-spectrum antibiotics is an important contributor to the rising rates of antimicrobial drug resistance worldwide. Drug utilization evaluation studies and pharmacists’ interventions are vital to assess, develop, and promote the rational use of antibiotics. This drug utilization study aimed to evaluate the current utilization practice of piperacillin/tazobactam in a hospital setting and assess the impact of pharmacist intervention in improving its appropriate use. (2) Methodology: In this study, we used a retrospective cohort and a prospective cohort, a cross-sectional, observational method. It included a retrospective (Cycle A/pre-intervention-CycA) phase followed by an educational interventional phase conducted by the pharmacists. During the 2 months of educational intervention, pharmacists used several methods, including workshops, lectures, oral presentations, and the development and reinforcement of clinical pathways to promote the judicious use of piperacillin/tazobactam. This was followed by a prospective (Cycle B/post-intervention-CycB) phase to improve piperacillin/tazobactam usage appropriateness. The appropriateness criteria for this drug utilization evaluation were established based on antimicrobial guidelines, the published literature, the institutional antibiogram, consultation from the antimicrobial stewardship committee, and the product monograph (Tazocin). The appropriateness of CycA and CycB patients was compared using the measurable elements, including indication and dose based on renal function, timely order for cultures, de-escalation, and use of extended infusion protocol. (3) Results: The study population comprised 100 patients in both CycA and CycB. The mean age of the patients was 66.28 ± 16.15 and 67.35 ± 17.98, and the ratios of men to women were found to be 49:51 and 61:39 in CycA and CycB, respectively. It was observed that inappropriate usage was high in CycA patients, and the appropriateness was improved in CycB patients. A total of 31% of inappropriate empirical broad-spectrum use was found in CycA, and it was reduced to 12% in CycB patients. The transition of appropriateness was observed in all measurable criteria, which includes the optimized dose according to the renal function (CycA = 49% to CycB = 94%), timely bacterial culture orders (CycA = 47% to CycB = 74%), prompt de-escalation (CycA = 31% to CycB = 53%), and adherence to extended infusion institutional guidelines (CycA = 34% to CycB = 86%). (4) Conclusions: The study highlighted important aspects of inappropriate piperacillin/tazobactam use. This can be considerably improved by proper education and timely interventions based on the pharmacists’ vigilant approach. The study results emphasized the need for surveillance of piperacillin/tazobactam usage by conducting similar drug utilization evaluations and practice to improve quality and safety in healthcare organizations globally.

## 1. Introduction

Drug utilization evaluation (DUE) is an efficient and systematic method to ensure the medication is used appropriately, safely, and effectively. By using a selective criteria-based approach, DUE can be used to identify the possible problem with drug use and if an intervention is needed to optimize therapeutic efficacy. For antimicrobial agents in particular, DUE can help reduce antimicrobial resistance and the risk of adverse events [1,2]. Bacteria with a high burden of multi-drug resistance are recognized as a major public health concern worldwide. Mortality from antimicrobial resistance (AMR) is estimated to be approximately 700,000 patients per year globally, with a predicted 10 million deaths by 2050 [3,4,5]. Fourth-generation cephalosporins, piperacillin/tazobactam, and carbapenems are the broadest-spectrum and most expensive antimicrobials and are a cornerstone empirical therapy for hospital-acquired severe infections. Therefore, the increasing threat of antimicrobial resistance due to the inappropriate use of these broad-spectrum antibiotics warrants the development of AMR reduction strategies [6,7]. Also, the use of broad-spectrum antibiotics can have clinically significant adverse effects, such as infection with multi-drug resistant bacteria, *Clostridium difficile*, and end-organ dysfunction [8].

Piperacillin/tazobactam is a combination of β-lactam/β-lactamase inhibitors. It has a broad spectrum of antibacterial activity against several microorganisms, including Gram-positive, anaerobic, and Gram-negative microorganisms, including many multidrug-resistant strains of *Pseudomonas aeruginosa* and *Enterobacteriaceae* species [9]. Piperacillin/tazobactam is useful against a variety of respiratory infections, intra-abdominal, skin, and soft tissue infections, febrile neutropenia, and bloodstream infections (BSI) [10,11,12].

Antimicrobial stewardship programs (ASPs) are a multidisciplinary quality improvement program composed of members that include physicians, pharmacists, infection prevention specialists, nurses, epidemiologists, microbiologists, information technology staff, and leadership. ASPs have proven to be effective in optimizing infection control measures, including decreasing infection rates and treatment failures [13]. DUE is an important tool for ASPs as it can be used to identify patterns of inappropriate prescribing. It is also an effective method to gain insight into the underlying causes of improper antimicrobial use, providing an opportunity to address the issue. Given the significance of the judicious use of broad-spectrum antibiotics, such as piperacillin/tazobactam, a DUE was conducted at Johns Hopkins Aramco Healthcare hospital in Saudi Arabia. The study included a retrospective analysis followed by clinical interventions by pharmacists to improve its utilization. This was followed by a prospective audit to measure improvement and further identify continuous measures to optimize antibiotic use.

## 2. Results

The study population comprised 100 patients in both CycA and CycB. Basic characteristics of the study population are illustrated in Table 1. The mean ages of the patients were 66.28 ± 16.15 and 67.35 ± 17.98 in CycA and CycB, respectively. The ratios of men to women in CycA and CycB were found to be 49:51 and 61:39, respectively.

The data show variations in the percentage of patients across different age groups between CycA and CycB (Table 2). In CycA, the highest percentage of patients is in the age group of 60–80 years (53%), followed by patients in the age group of 40–60 years (20%). In CycB, the highest percentage of patients are also in the age group of 60–80 years (49%), followed by patients in the age group of 40–60 years (16%). Interestingly, in CycA, the age group of 18–40 years accounted for only 6% of patients, which increased to 9% in CycB. Conversely, the age group of 80+ years accounted for 20% of patients in CycA, which increased to 26% in CycB. The Wilcoxon signed ranks test was used to compare the SCr (serum creatinine) levels between CycA and CycB. The test results presented a Z-value of −0.953 and the associated two-tailed *p*-value 0.341. The data did not provide enough evidence to conclude that the SCr levels are significantly different between CycA and CycB. Mean serum creatinine in CycA was 1.343 mg/dL while CycB was 1.083 mg/dL. Similarly, average creatinine clearance was 67.04 mL/min for CycA and 71.8 mL/min in CycB. Similarly, a Wilcoxon signed-rank test was conducted to compare the CrCl (creatinine clearance) levels between CycA and CycB. The test results show that the Z-value is −0.536 and the associated two-tailed *p*-value is 0.592. There is no significant difference between the CrCl levels in CycA and CycB. In other words, the data do not provide enough evidence to conclude that the CrCl levels are significantly different between CycA and CycB.

No significant differences were noted between the incidences of hypertension, chronic obstructive pulmonary disease, coronary artery disease, cancer, and chronic kidney disease (Table 2).

**Figure 1:**
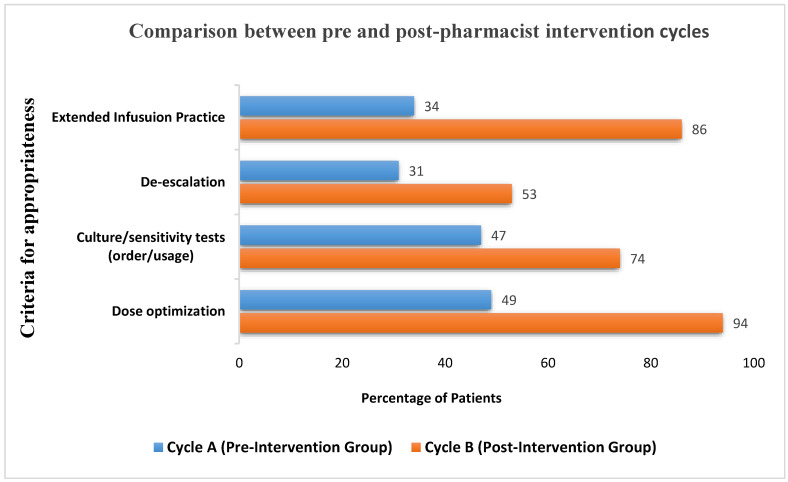
Difference in appropriateness criteria between pre- and post-pharmacist intervention groups.

The parameters for therapy appropriateness for CycA and CycB are illustrated in Figure 1. The measured elements included appropriate indication for empirical use, appropriate dosage based on indication and renal function, while culture/sensitivity was ordered at the appropriate time to guide antimicrobial therapy, de-escalation, and switch to extended infusion based on institutional guidelines.

The Lexi-Comp (JHAH’s) organizational drug reference database was used to determine if the piperacillin/tazobactam prescribed dosage was correct based on indication and the patients’ renal function. During CycA, 49% met the criteria, while CycB showed a major improvement (94%). McNemar’s test was applied to compare dosing appropriateness between CycA and CycB. The test results presented the chi-square statistic of 36.98, with an asymptotic significance level of 0. The low *p*-value indicated a significant difference in dosing appropriateness between CycA and CycB. Hence, the impact was deemed significant.

Based on microbiology cultures, each case was followed up for de-escalation. Compared to CycA (31%), a greater number of cases in CycB (53%) noted significant improvement. De-escalation based on the McNemar test with a sample size of 100, a chi-square value of 4.78, and a *p*-value of 0.029 was obtained. This indicated a statistically significant difference between adherence to the de-escalation protocol.

Significant improvements were also seen in culture/sensitivity ordered in a timely manner to guide antimicrobial therapy, with an increase from 47% in CycA to 74% in CycB. Also, in CycA, only 34% of the patients were transitioned from intermittent to extended infusion. However, after the educational intervention, 86% of the patients were switched to extended infusion in a timely manner. A statistically significant association between external infusion rates and culture and sensitivity orders was not established with chi-square test results.

## 3. Discussion

This study on piperacillin/tazobactam observed that pharmacists’ involvement through educational interventions helped to improve the appropriateness of use, resulted in increased culture/sensitivity tests being ordered in a timely manner, improved the de-escalation rate, and saw a higher level of utilization of the extended infusion protocol. Each of these findings are explained one by one here.

### 3.1. Inappropriate Use: The Spectrum of Activity Was Too Broad

One of the most significant reasons identified for inappropriate usage was a broad-spectrum use of piperacillin/tazobactam. As noted, it was prescribed by internal medicine empirically (determined from an indication selected by the prescriber in EHR) for fever of unknown origin, uncomplicated cholecystitis, appendicitis, and suspected skin and soft tissue infections of mild severity. In addition, it was also noted that it was misused as an empiric treatment for uncomplicated community-acquired urinary tract infections. One of the most effective way to align clinical practice with guideline recommendations is to develop clinical pathways. Clinical pathways have proven to reduce variation in clinical practice while enabling the provision of safe, evidence-based care to improve patient outcomes [14]. Lack of use and adherence to institutional clinical pathways, such as febrile neutropenia and community-acquired pneumonia, by the prescribers was noted as an important concern contributing to many instances of inappropriate empirical selection of piperacillin/tazobactam. Due to the pharmacists’ interventions, the inappropriate use of piperacillin/tazobactam as a result of its initial empirical selection improved from CycA (*n* = 34) to CycB (*n* = 21). A higher number of patients were prescribed piperacillin/tazobactam empirically to provide broad-spectrum coverage in CycA (31%). The appropriateness of this empiric therapy was notably incorrect, as the spectrum of activity for the associated indication was too broad. Due to the pharmacists’ intervention, the use of targeted therapy was improved, and inappropriate empiric coverage decreased during CycB (19%). However, the continued existence of inappropriate empirical selection of piperacillin/tazobactam in CycB has led to the recognition of an important deficiency of institution-specific guidelines for the use of piperacillin/tazobactam. Also, it drew special attention to the need to enact a method to measure the compliance of prescribers with adherence to clinical pathways developed at JHAH for various infectious diseases.

### 3.2. Dosage Is Appropriate Based on Indication and Renal Function

Epidemiological studies have shown an association between antimicrobial consumption and the emergence and spread of bacterial resistance, particularly for intensive care unit patients [15,16]. Treating resistant infections is highly challenging and sometimes impossible. Administering the recommended antibacterial dose to the patient is one effective mechanism to achieve maximum antibiotic exposure, reducing the potential of antibiotic resistance development. Using pharmacodynamics principles to develop appropriate dosing regimens which treat not only the isolated bacteria, but also its most resistant subpopulation, can help prevent the emergence of further resistant infections [17]. In addition, the inappropriate use of antimicrobials in patients with chronic kidney disease is a major cause of hospitalization, increased length of stay, higher therapeutic costs, adverse drug reactions, toxicities, therapeutic failure, and mortality. Appropriate and timely dosage modifications in patients with renal dysfunction are also important, as otherwise normal doses can become augmented or diminished in these patients due to alterations in the pharmacokinetic properties of several medications. Studies from China showed that antibiotics-related dosage errors in chronic kidney disease (CKD) patients occurred at rates of between 38.8–60.3%. The economic burden from CKD complications remains high, with a strong correlation, around 25–77%, to inappropriate dosing adjustments. One important challenge faced by clinicians is the marked variation in renal dose adjustment recommendations among different guidelines. Therefore, an intervention by a clinician or pharmacist is essential to apply the recommendation safely. Clinical pharmacists can, therefore, play an important role in providing optimal pharmaceutical care for in patients with renal dysfunction [18,19]. In this study the low *p*-value indicated a significant difference in dosing appropriateness between Cycle A and Cycle B. Hence, the impact was deemed significant. A retrospective chart review study from Lebanon concluded that piperacillin/tazobactam was the most frequently prescribed drug (30.6%) without renal dose adjustment in hospital settings [20]. Also, several studies indicate a higher frequency of renal disorders in patients receiving piperacillin/tazobactam therapy with a significantly slower renal clearance in patients with severe renal failure. It is a significant risk factor for acute kidney injury in patients in the mean age group of 70. At higher doses, piperacillin/tazobactam tends to saturate the renal tubules, therefore, prolonging its elimination. In addition, comparative studies have shown that the renal recovery rate in patients receiving piperacillin/tazobactam was lower than that in critically ill patients treated with other antibiotics. For example, patients who received a combination of vancomycin with piperacillin/tazobactam had a higher incidence of nephrotoxicity (16.3%) vs. vancomycin alone (8.08%) [21,22].

CycA of the current study provides important insights into the inappropriate current use of piperacillin/tazobactam vs. CycB, where significant improvement is noted due to pharmacist intervention. The Lexi-Comp online drug information database provided the guidelines used to evaluate the appropriateness of dosing, based on indication and renal dose adjustment.

### 3.3. Culture/Sensitivity/De-Escalation

Inappropriate and suboptimal use of antibiotics may lead to multiple disadvantages, such as an increased length of stay, spread or emergence of antimicrobial resistance, and mortality. De-escalation is one of the essential tools for optimizing the rational use of antibiotics [23]. Therefore, the use of cultures and sensitivity reports is clinically essential to help narrow antibiotic therapy and carry out effective de-escalation. The literature review has highlighted that the prevalence of de-escalation varies and ranges anywhere from 10% to 70% [24,25,26,27,28]. Also, multiple studies have shown evidence that antibiotic de-escalation is a very safe, effective, and clinically appropriate mechanism to avoid unnecessary antibiotic use that would promote the development of resistance [29,30,31]. The most common reasons for a poor de-escalation rate reported include either the fear of changing antibiotics considering the severity of the disease, clinical judgment, available personnel, or simply the habit of keeping the antibiotic despite symptomatic improvement [25,27,28,32].

As a result of pharmacy intervention, a significant improvement was noted from CycA (*n* = 47) to CycB (*n* = 74) for prompt ordering of culture and sensitivity testing and its usage to guide antimicrobial therapy. Additionally, a 22% improvement was noted in de-escalation between CycA and CycB. Although the pharmacist interventions improved the de-escalation rate in CycB, it remained low and was noted as a focus area of concern requiring more aggressive multidisciplinary intervention in the future.

### 3.4. Extended Infusion

As a result of seriously emerging antimicrobial resistance to Gram-negative and Gram-positive bacteria, antibiotic stewardship programs are putting a lot of emphasis on implementing antibiotic regimens based on pharmacodynamics. Similar to other beta-lactam antibiotics, piperacillin/tazobactam exhibits time-dependent killing. The time (T) that the unbound fraction (f) of the drug remains above the minimum inhibitory concentration (MIC) is the pharmacokinetics/pharmacodynamics (PK/PD) index of choice (fT > MIC). Prolonging the infusion time via extended infusion (EI) and maintaining the beta-lactam levels above the MIC for a percentage of dosing interval ensures near maximal bactericidal effect and improves the probability of target attainment [33,34]. Compared to the traditional intermittent (30 min) infusion, the extended infusion in critically ill patients with severe sepsis has decreased mortality, improved outcomes, and lowered the odds of *C. difficile* infection [35,36,37]. A cost-effectiveness study by Naiim et al. showed that piperacillin/tazobactam extended infusion was superior to intermittent infusion the regarding cost-effectiveness ratio ($1835.41 and $1914.09/expected success, respectively). Additional cost analysis studies by Brunetti et al. and Bao et al. similarly demonstrated that using extended infusion of piperacillin/tazobactam is associated with significant cost savings ($316.04 in the intermittent infusion group vs. $146.66 in the EI group). This was mainly attributed to a decrease in the duration of antimicrobial therapy until treatment success [36,38].

Based on the superior clinical and economic outcomes reported, the JHAH antimicrobial stewardship committee has also developed an institutionalized extended infusion piperacillin/tazobactam protocol. A significant component of CycB included pharmacist intervention to improve adherence to this protocol which has implemented the use of prolonged piperacillin/tazobactam infusions for patients with suspected infections or treatment for confirmed infections caused by pathogens with high antimicrobial MICs (piperacillin/tazobactam has an MIC of 16 mg/L). The pharmacist interchanged all intermittent infusion orders after an initial 30 min bolus dose with extended infusion (over 4 h) as outlined in the protocol unless they met one of the following exception criteria, such as pediatric population (<18 years old) or hemodialysis and peritoneal dialysis patients, etc. Due to the pharmacist intervention, the adherence to the extended infusion protocol was observed to be significantly improved in CycA (*n* = 34) vs. CycB (*n* = 86). This can, therefore, be linked to clinical and economic benefits already established in the literature for extended infusion administration of piperacillin/tazobactam. To the best of our knowledge, this is the first drug utilization evaluation in Saudia Arabia, where a prospective audit was actively conducted by the pharmacists to continuously improve the empirical piperacillin/tazobactam utilization. A single center-based study and the minimal number of patients enrolled is a minor limitation of this study. While an institutional guideline for the use of piperacillin/tazobactam is not available, this limitation was overcome by utilizing a hospital antibiogram and international antimicrobial guidelines. It is recommended to compare the results with multiple centers in order to increase the reliability of this study’s findings.

## 4. Methodology

### 4.1. Study Setting and Design

This study was conducted at Johns Hopkins Aramco Healthcare (JHAH), a 483-bed hospital in Saudi Arabia, including major departments and services, such as medical, surgical, hematology, oncology, intensive care units, neonatal, pediatrics, laboratory, and pharmacy. The DUE criteria and process for piperacillin/tazobactam was approved by the Pharmacy and Therapeutics committee. The study period covered the months of January 2022 through March 2023. The study design included three total periods, Cycle A (pre-intervention/retrospective-CycA), educational intervention, and Cycle B (post-intervention/prospective-CycB). The appropriateness of piperacillin/tazobactam use was evaluated according to the recommendation of the Infectious Disease Society of America (IDSA) guidelines, institutional antibiogram, and the guidance from antimicrobial stewardship clinical pharmacists. During the CycA period (January 2022 until October 2022), an extensive chart review of 100 patients who received piperacillin/tazobactam was conducted.

The next period of the study consisted of 2 months of educational intervention carried out in November and December of 2022. During this period, several educational interventions were conducted in correspondence with an infectious disease consultant and an infectious disease clinical pharmacist to address the problems identified with the use of piperacillin/tazobactam. These included sharing educational materials and holding meetings (oral presentations, lectures, training workshops) for both clinicians and pharmacists. Upon order verification, pharmacists were mandated to document all interventions using an electronic I-vent in the electronic health record (EHR) system. This enabled the creation of a system with retrievable information using electronic methods to document improvement in piperacillin/tazobactam use. In addition, this period included a review of institutional clinical pathways, such as community-acquired pneumonia and febrile neutropenia pathways. This ensured that the clinical pathways were being utilized correctly and in line with the IDSA-approved guidelines. The findings from the CycA period were shared during lectures and workshops to emphasize the need for more effective and rational use of piperacillin/tazobactam. Also, reminders and other communication strategies, such as the EPIC “secure chat” system, were used to prompt healthcare providers to recall information and discuss patients’ piperacillin/tazobactam treatment regimen. The goal of this period was to promote standardization in the management and clinical use of piperacillin/tazobactam.

The final part of the study (CycB) was conducted on 100 patients from January 2023 until March 2023. A team of clinical pharmacists monitored all patients started on piperacillin/tazobactam using a report created in EHR. Based on DUE criteria for empirical use, as well as culture and sensitivity results, a de-escalation from piperacillin/tazobactam to another antimicrobial was carried out in a timely manner. Also, in some instances, piperacillin/tazobactam was discontinued after consulting with the prescribing physician. The overall appropriate use was measured.

### 4.2. Data Collection

The patient’s charts were reviewed by accessing the EPIC (EHR) system; we extracted the required data and exported these to an Excel sheet. The following data were collected during the retrospective analysis: patient demographics, empirical indication for piperacillin/tazobactam use (selected in EHR by the prescribing physician), dose and duration of piperacillin/tazobactam therapy, number of doses, culture and sensitivity results, concomitant antibiotics, renal function (serum creatinine, creatinine clearance, hemodialysis, or peritoneal dialysis), the total length of stay (LOS) in the hospital, presence of other medical conditions including hypertension, diabetes mellitus (DM), chronic obstructive pulmonary disease (COPD), asthma, coronary artery disease (CAD), cancer, chronic kidney disease/hemodialysis, acute kidney injury, or concomitant COVID-19 infection, and use of intermittent infusion (infusion lasting 30–60 min) vs. extended infusion (infusion lasting 4 h), based on the approved institutional guideline. Analysis of the appropriateness of piperacillin/tazobactam indication, dose, frequency, and duration of therapy was based on the available antibiotic stewardship guidelines (ASG) of the Infectious Diseases Society of America (IDSA).

### 4.3. Inclusion and Exclusion Criteria

Patients ≥ 18 years’ old who started on piperacillin/tazobactam during hospitalization were included. Patients with a single dose of piperacillin/tazobactam in the emergency department, peri-operative prophylaxis, and pregnant women were excluded.

### 4.4. Statistical Analysis 

The data retrieved from Epic Hyperspace were pooled in Microsoft Excel and analyzed using SPSS version 26.0 (IBM SPSS Inc., Chicago, IL, USA). Descriptive statistics were used, and the data were presented as frequencies and percentages. The mean and standard deviation of CycA and CycB were calculated. Different statistical tests were used to compare the outcomes between CycA and CycB. A probability of less than 0.05 was considered to be significant.

## 5. Conclusions

The first period of the study played a major role in detecting inappropriate use of piperacillin/tazobactam with regard to broad-spectrum empirical use, dosage based on indication and patients’ renal function, de-escalation, and adherence to an extended infusion protocol. The pharmacist intervention, including educational efforts, improved piperacillin/tazobactam overall utilization in the subsequent phase, and influenced a change during the prospective audit. This DUE process, therefore, laid out a model for continuous improvement in the future to create an effective and rational system for using antibiotics.

## Figures and Tables

**Table 1:** Basic characteristics of the study population.

Parameters		Cycle A (*n* = 100)	Cycle B (*n* = 100)
Age and gender	Mean age with standard deviation ^#^	66.28 ± 16.15	67.35 ± 17.98
	Men to women proportion ^>^	49:51	61:39
Creatinine	Mean and SD of serum creatinine	1.34 mg/dL SD: 1.44 mg/dL	1.083 mg/dL SD: 0.95 mg/dL
	Mean and SD of creatinine clearance	67.04 mL/min SD: 41.83 mL/min	71.8 mL/min SD: 52.69 mg/dL
Indications (in numbers)	Bacterial pneumonia	47%	50%
	Cholecystitis	3%	1%
	Diabetic foot infection	5%	6%
	Febrile neutropenia	4%	0%
	Fever	3%	1%
	Intra-abdominal abscess	2%	4%
	Peritonitis	1%	4%
	Sepsis	9%	9%
	Skin and skin structure infection	17%	9%
	Urinary tract infection	3%	3%
	Others *	6%	13%

# *p*-value 0.668; > *p*-value 0.088.* Other indications include (CycA %: CycB %) abdominal infection (0:2), abscess tonsillitis (0:2), appendicitis (1:1), aspiration pneumonia (1:1), bone infection (0:1), cholangitis (0:2), CLABSI (0:1), COPD exacerbation with history of *Pseudomonas* (1:0), enterocolitis (1:0), and febrile neutropenic presumed infection (0:1). CLABSI: central line-associated bloodstream infection. COPD: chronic obstructive pulmonary disease; SD: standard deviation.

**Table 2:** Tests of association between variables among the subjects (Pearson chi-square test).

Variables	Percentage of Patients in Each Cycle (*n* = 100)		Value	Significance
	Cycle A	Cycle B		
Chronic kidney disease	27	23	1.399	0.237
Hypertension	66	47	0.701	0.402
Coronary artery disease	17	14	1.121	0.29
Chronic obstructive pulmonary disease	19	10	0.007	0.932

## Data Availability

The data will be made available by contacting the corresponding using the provided email. Restrictions are applicable according to the IRB of the organization.

## References

[1] Korai U., Naqvi G.R., Zafar F., Ali H., Naeem S., Alam N., Saeed R., Farooqi S., Hussain T. (2019). Drug utilization evaluation of Piperacillin/Tazobactam: A prospective and cross sectional investigation in tertiary care setup. Pak. J. Pharm. Sci..

[2] Samilski J.A.E., Lau T.T.Y., Elbe D.H.T., Aulakh A.K., Lun E.M.C. (2012). Drug use evaluation of moxifloxacin (avelox) using a hand-held electronic device at a canadian teaching hospital. Pharm. Ther..

[3] Lieberman J.M. (2003). Appropriate antibiotic use and why it is important: The challenges of bacterial resistance. Pediatr. Infect. Dis. J..

[4] Tacconelli E., Carrara E., Savoldi A., Harbarth S., Mendelson M., Monnet D.L., Pulcini C., Kahlmeter G., Kluytmans J., Carmeli Y. (2018). Discovery, research, and development of new antibiotics: The WHO priority list of antibiotic-resistant bacteria and tuberculosis. Lancet Infect. Dis..

[5] Da Silva J.B., Espinal M., Ramón-Pardo P. (2020). Antimicrobial resistance: Time for action. Rev. Panam. Salud. Publica.

[6] Raveh D., Muallem-Zilcha E., Greenberg A., Wiener-Well Y., Schlesinger Y., Yinnon A.M. (2006). Prospective drug utilization evaluation of three broad-spectrum antimicrobials: Cefepime, piperacillin-tazobactam and meropenem. QJM Int. J. Med..

[7] Alsaleh N.A., Al-Omar H.A., Mayet A.Y., Mullen A.B. (2020). Evaluating the appropriateness of carbapenem and piperacillin-tazobactam prescribing in a tertiary care hospital in Saudi Arabia. Saudi Pharm. J..

[8] Wiens J., Snyder G.M., Finlayson S., Mahoney M.V., Celi L.A. (2018). Potential Adverse Effects of Broad-Spectrum Antimicrobial Exposure in the Intensive Care Unit. Open Forum Infect. Dis..

[9] Bryson H.M., Brogden R.N. (1994). Piperacillin/tazobactam. A review of its antibacterial activity, pharmacokinetic properties and therapeutic potential. Drugs.

[10] Freifeld A.G., Bow E.J., Sepkowitz K.A., Boeckh M.J., Ito J.I., Mullen C.A., Raad I.I., Rolston K.V., Young J.-A.H., Wingard J.R. (2011). Clinical practice guideline for the use of antimicrobial agents in neutropenic patients with cancer: 2010 update by the infectious diseases society of america. Clin. Infect. Dis..

[11] Evans L., Rhodes A., Alhazzani W., Antonelli M., Coopersmith C.M., French C., Machado F.R., Mcintyre L., Ostermann M., Prescott H.C. (2021). Surviving sepsis campaign: International guidelines for management of sepsis and septic shock 2021. Intensive Care Med..

[12] Mazuski J.E., Tessier J.M., May A.K., Sawyer R.G., Nadler E.P., Rosengart M.R., Chang P.K., O’Neill P.J., Mollen K.P., Huston J.M. (2017). The Surgical Infection Society Revised Guidelines on the Management of Intra-Abdominal Infection. Surg. Infect..

[13] Ha D.R., Haste N.M., Gluckstein D.P. (2019). The Role of Antibiotic Stewardship in Promoting Appropriate Antibiotic Use. Am. J. Lifestyle Med..

[14] Busse R., Klazinga N., Panteli D., Quentin W. (2019). Improving Healthcare Quality in Europe: Characteristics, Effectiveness and Implementation of Different Strategies.

[15] Bell B.G., Schellevis F., Stobberingh E., Goossens H., Pringle M. (2014). A systematic review and meta-analysis of the effects of antibiotic consumption on antibiotic resistance. BMC Infect. Dis..

[16] Fishman N. (2006). Antimicrobial stewardship. Am. J. Infect. Control.

[17] Roberts J.A., Kruger P., Paterson D.L., Lipman J. (2008). Antibiotic resistance—What’s dosing got to do with it?. Crit. Care Med..

[18] Hassan Z., Ali I., Ullah A.R., Ahmed R., Zar A., Ullah I., Rehman S., Khan A.U., Ullah R., Hanif M. (2021). Assessment of Medication Dosage Adjustment in Hospitalized Patients With Chronic Kidney Disease. Cureus.

[19] Fahimi F., Emami S., Rashid-Farokhi F. (2012). The Rate of Antibiotic Dosage Adjustment in Renal Dysfunction. Iran. J. Pharm. Res. IJPR.

[20] Chahine B. (2022). Antibiotic dosing adjustments in hospitalized patients with chronic kidney disease: A retrospective chart review. Int. Urol. Nephrol..

[21] Morimoto T., Nagashima H., Morimoto Y., Tokuyama S. (2017). Frequency of Acute Kidney Injury Caused by Tazobactam/Piperacillin in Patients with Pneumonia and Chronic Kidney Disease: A Retrospective Observational Study. Yakugaku Zasshi.

[22] Kadomura S., Takekuma Y., Sato Y., Sumi M., Kawamoto K., Itoh T., Sugawara M. (2019). Higher incidence of acute kidney injury in patients treated with piperacillin/tazobactam than in patients treated with cefepime: A single-center retrospective cohort study. J. Pharm. Health Care Sci..

[23] Campion M., Scully G. (2018). Antibiotic Use in the Intensive Care Unit: Optimization and De-Escalation. J. Intensive Care Med..

[24] Silva B.N.G., Andriolo R.B., Atallah A.N., Salomão R. (2013). De-escalation of antimicrobial treatment for adults with sepsis, severe sepsis or septic shock. Cochrane Database Syst. Rev..

[25] Garnacho-Montero J., Gutiérrez-Pizarraya A., Escoresca-Ortega A., Corcia-Palomo Y., Fernández-Delgado E., Herrera-Melero I., Ortiz-Leyba C., Márquez-Vácaro J.A. (2014). De-escalation of empirical therapy is associated with lower mortality in patients with severe sepsis and septic shock. Intensive Care Med..

[26] Leone M., Bechis C., Baumstarck K., Lefrant J.-Y., Albanèse J., Jaber S., Lepape A., Constantin J.-M., Papazian L., Bruder N. (2014). De-escalation versus continuation of empirical antimicrobial treatment in severe sepsis: A multicenter non-blinded randomized noninferiority trial. Intensive Care Med..

[27] Masterton R.G. (2011). Antibiotic de-escalation. Crit. Care Clin..

[28] Alvarez-Lerma F., Alvarez B., Luque P., Ruiz F., Dominguez-Roldan J.-M., Quintana E., Sanz-Rodriguez C. (2006). Empiric broad-spectrum antibiotic therapy of nosocomial pneumonia in the intensive care unit: A prospective observational study. Crit. Care.

[29] De Waele J.J., Ravyts M., Depuydt P., Blot S.I., Decruyenaere J., Vogelaers D. (2010). De-escalation after empirical meropenem treatment in the intensive care unit: Fiction or reality?. J. Crit. Care.

[30] Donaldson A.D., Barkham T. (2010). De-escalation for amoxicillin-susceptible *Escherichia coli*: Easier said than done. J. Hosp. Infect..

[31] Rello J., Vidaur L., Sandiumenge A., Rodríguez A., Gualis B., Boque C., Diaz E. (2004). De-escalation therapy in ventilator-associated pneumonia. Crit. Care Med..

[32] Morel J., Casoetto J., Jospé R., Aubert G., Terrana R., Dumont A., Molliex S., Auboyer C. (2010). De-escalation as part of a global strategy of empiric antibiotherapy management. A retrospective study in a medico-surgical intensive care unit. Crit. Care.

[33] Dhaese S.A.M., Hoste E.A., De Waele J.J. (2022). Why We May Need Higher Doses of Beta-Lactam Antibiotics: Introducing the ‘Maximum Tolerable Dose’. Antibiotics.

[34] Abdul-Aziz M.H., Dulhunty J.M., Bellomo R., Lipman J., Roberts J.A. (2012). Continuous beta-lactam infusion in critically ill patients: The clinical evidence. Ann. Intensive Care.

[35] Fawaz S., Barton S., Nabhani-Gebara S. (2020). Comparing clinical outcomes of piperacillin-tazobactam administration and dosage strategies in critically ill adult patients: A systematic review and meta-analysis. BMC Infect Dis..

[36] Chan A.J., Lebovic G., Wan M., Chen Y., Leung E., Langford B.J., Seah J., Taggart L.R., Downing M. (2023). Impact of extended-infusion piperacillin-tazobactam in a Canadian community hospital. Infect. Med..

[37] Roberts J.A., Abdul-Aziz M.-H., Davis J.S., Dulhunty J.M., Cotta M.O., Myburgh J., Bellomo R., Lipman J. (2016). Continuous versus Intermittent β-Lactam Infusion in Severe Sepsis. A Meta-analysis of Individual Patient Data from Randomized Trials. Am. J. Respir. Crit. Care Med..

[38] Naiim C.M., Elmazar M.M., Sabri N.A., Bazan N.S. (2022). Extended infusion of piperacillin-tazobactam versus intermittent infusion in critically ill egyptian patients: A cost-effectiveness study. Sci. Rep..

